# Supplementary material for: Image-based, pooled phenotyping reveals multidimensional, disease-specific variant effects
Source: bioRxiv. 2025 Jul 5:2025.07.03.663081. Preprint. [Version 1] doi: 10.1101/2025.07.03.663081 (PMC12236469; doi:10.1101/2025.07.03.663081)
Supplement: 1 [file NIHPP2025.07.03.663081V1-supplement-1.pdf]

bioRxiv preprint doi: https://doi.org/10.1101/2025.07.03.663081; this version posted July 5, 2025. The copyright holder for this preprint (which was not certified by peer review) is the author/funder, who has granted bioRxiv a license to display the preprint in perpetuity. It is made available under aCC-BY 4.0 International license.

SUPPLEMENTAL FIGURES

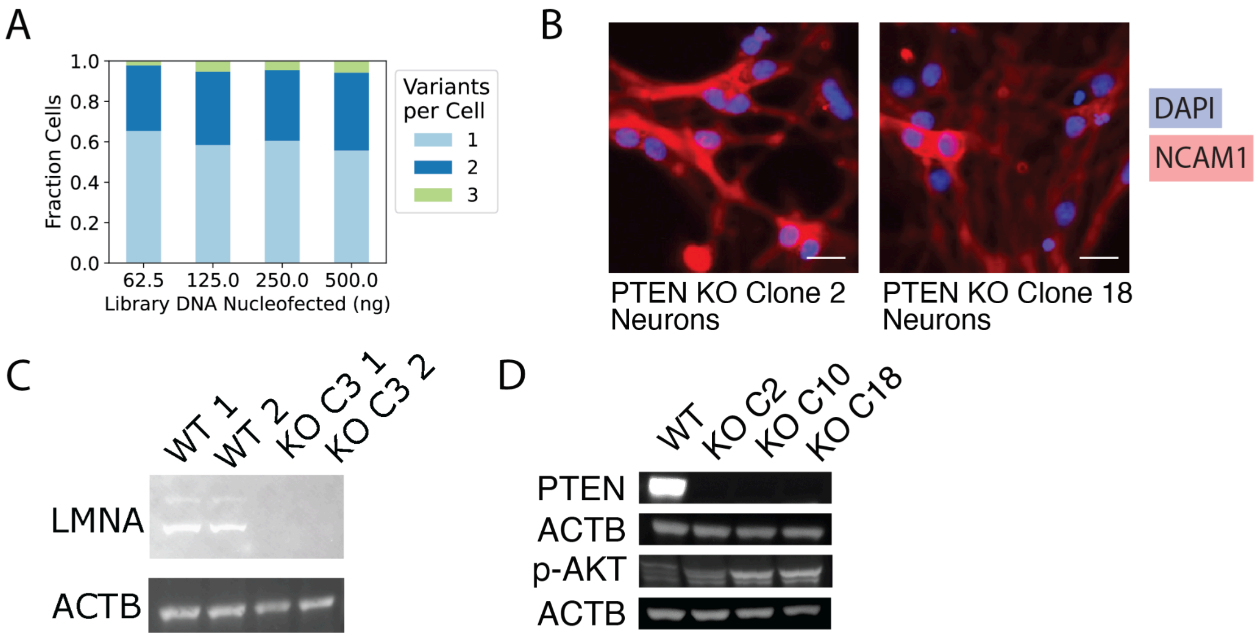

Supplementary Figure 1: *piggyBac* MOI titration and knockout Western blots

(A) Fraction of cells with 1, 2, or 3 integrations, determined by 4-base *in situ* sequencing, after co-transfection of *LMNA* VIS-seq library DNA at different quantities (in nanograms) with plasmid encoding Piggybac-ase (at a 4-fold lower mass).

(B) Imaging of day-7 neurons derived from clonal NGN2-inducible *PTEN* knockout lines stained for DAPI (blue) and NCAM1 (red). Scale bar indicates 20 µm.

(C) Western blot of U2OS cells showing parental line (left) and *LMNA* knockout lines (right) stained for lamin A protein.

(D) Western blot of NGN2-inducible iPS cells showing parental line (left) and *PTEN* knockout lines (right) stained for PTEN and phospho-AKT protein. Clonal lines C2 and C18 were used for *PTEN* VIS-seq experiments.

bioRxiv preprint doi: https://doi.org/10.1101/2025.07.03.663081; this version posted July 5, 2025. The copyright holder for this preprint (which was not certified by peer review) is the author/funder, who has granted bioRxiv a license to display the preprint in perpetuity. It is made available under aCC-BY 4.0 International license.

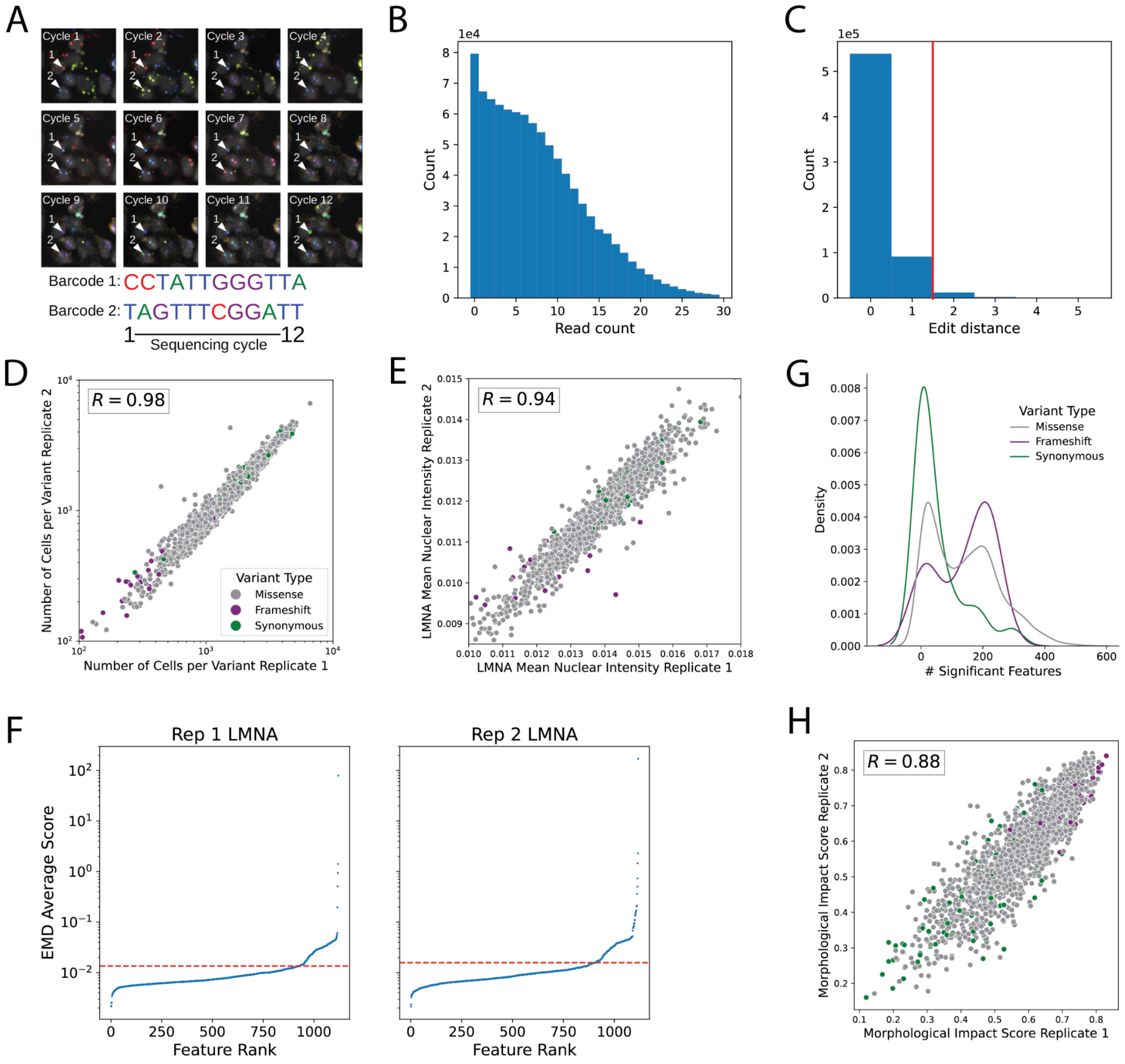

Supplementary Figure 2: *LMNA* VIS-seq replication and visualization

(A) 12-base pair barcode sequences on circular RNAs were read by *in situ* sequencing by synthesis in each cell, and then mapped to corresponding *LMNA* variants using a barcode-to-variant dictionary made by long-read sequencing. Example cells with reads in all 12 cycles are shown.

(B) Histogram of total number of 12-base pair reads per sequenced cell in single well of *LMNA* replicate 2 experiment.

(C) Edit distance between consensus cell-level 12-base pair read and nearest library barcode in a single well of *LMNA* replicate 2 experiment. Red line indicates that cells with edit distance < 2 were used if they matched to a unique barcode.

(D) Number of cells genotyped for each *LMNA* variant in both replicates of VIS-seq screen colored by variant type, with Pearson's r shown.

bioRxiv preprint doi: https://doi.org/10.1101/2025.07.03.663081; this version posted July 5, 2025. The copyright holder for this preprint (which was not certified by peer review) is the author/funder, who has granted bioRxiv a license to display the preprint in perpetuity. It is made available under aCC-BY 4.0 International license.

(E) Mean nuclear intensity in the mEGFP-lamin A channel (median over cells per variant) for variants in both replicates of VIS-seq screen colored by variant type as in (D), with Pearson's r shown.
(F) EMD reproducibility scores derived from 30 random partitions of wild-type *LMNA* expressing cells ranked by feature for replicate 1 (left) and replicate 2 (right). Low scores indicate high reproducibility. Threshold is drawn at 1.5 times the IQR added to the first quartile. Feature EMDs above this threshold in either replicate were removed in the feature selection step.
(G) Number of significant features for each variant class determined by KS-test against wild-type cells, colored by variant type. Significance thresholded by Bonferroni-corrected $p<0.001$.
(H) Morphological impact score of variants in both replicates of VIS-seq screen colored by variant type as in (D), with Pearson's r shown.

bioRxiv preprint doi: https://doi.org/10.1101/2025.07.03.663081; this version posted July 5, 2025. The copyright holder for this preprint (which was not certified by peer review) is the author/funder, who has granted bioRxiv a license to display the preprint in perpetuity. It is made available under aCC-BY 4.0 International license.

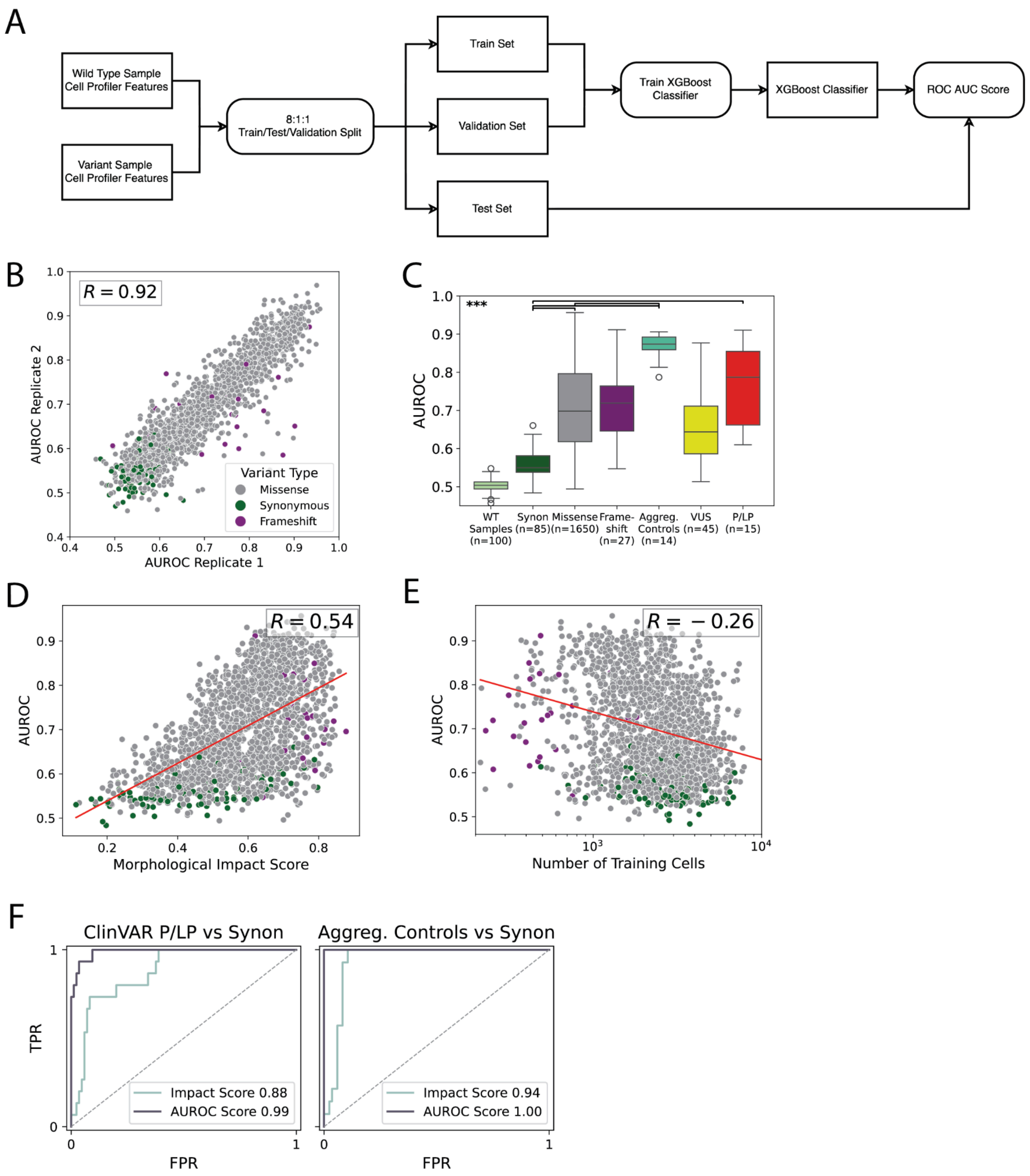

Supplementary Figure 3: Training models to distinguish profiled *LMNA* variants from WT

(A) Flow-chart describing training binary classifiers for each variant to distinguish single cell images of that variant from corresponding WT images. For each variant as well as bootstraps of 1000 WT cells, an AUROC score summarizing classifier performance was computed on a test set of single cells. 0.5 indicates random classifier performance and 1 indicates perfect discrimination between variant and WT single cells. For a full description, see Methods.

bioRxiv preprint doi: https://doi.org/10.1101/2025.07.03.663081; this version posted July 5, 2025. The copyright holder for this preprint (which was not certified by peer review) is the author/funder, who has granted bioRxiv a license to display the preprint in perpetuity. It is made available under aCC-BY 4.0 International license.

(B) AUROC of variants from both replicates of VIS-seq screen colored by variant type, with Pearson's r shown.
(C) AUROC scores for *LMNA* variants are plotted by variant type: missense, frameshift, or synonymous (Synon). 100 bootstrapped samples of WT variants are also shown for comparison. Scores are also shown for variants with >15% aggregated cells in HEK 293T previously measured by Anderson *et al*[39]. Lastly, AUC scores are plotted for ClinVar classifications of: variant of uncertain significance (VUS) and likely pathogenic/pathogenic (LP/P). *** indicates Mann-Whitney p-value < 0.001.
(D) Scatterplot showing the AUROC score for each variant against the morphological impact score of that variant. Variants are colored by variant type. Best fit line shown in red.
(E) Scatterplot showing the AUROC score for each variant against the number of training cells used for that variant. Variants are colored by variant type. Best fit line shown in red.
(F) Receiver operating characteristic (ROC) curves are plotted for univariate zero-shot models trained on VIS-seq *LMNA* variant morphological impact or AUROC scores (solid lines, see Methods for details) predicting ClinVar pathogenicity or aggregation[39] Area under the curve (AUC) scores are also shown for each model.

bioRxiv preprint doi: https://doi.org/10.1101/2025.07.03.663081; this version posted July 5, 2025. The copyright holder for this preprint (which was not certified by peer review) is the author/funder, who has granted bioRxiv a license to display the preprint in perpetuity. It is made available under aCC-BY 4.0 International license.

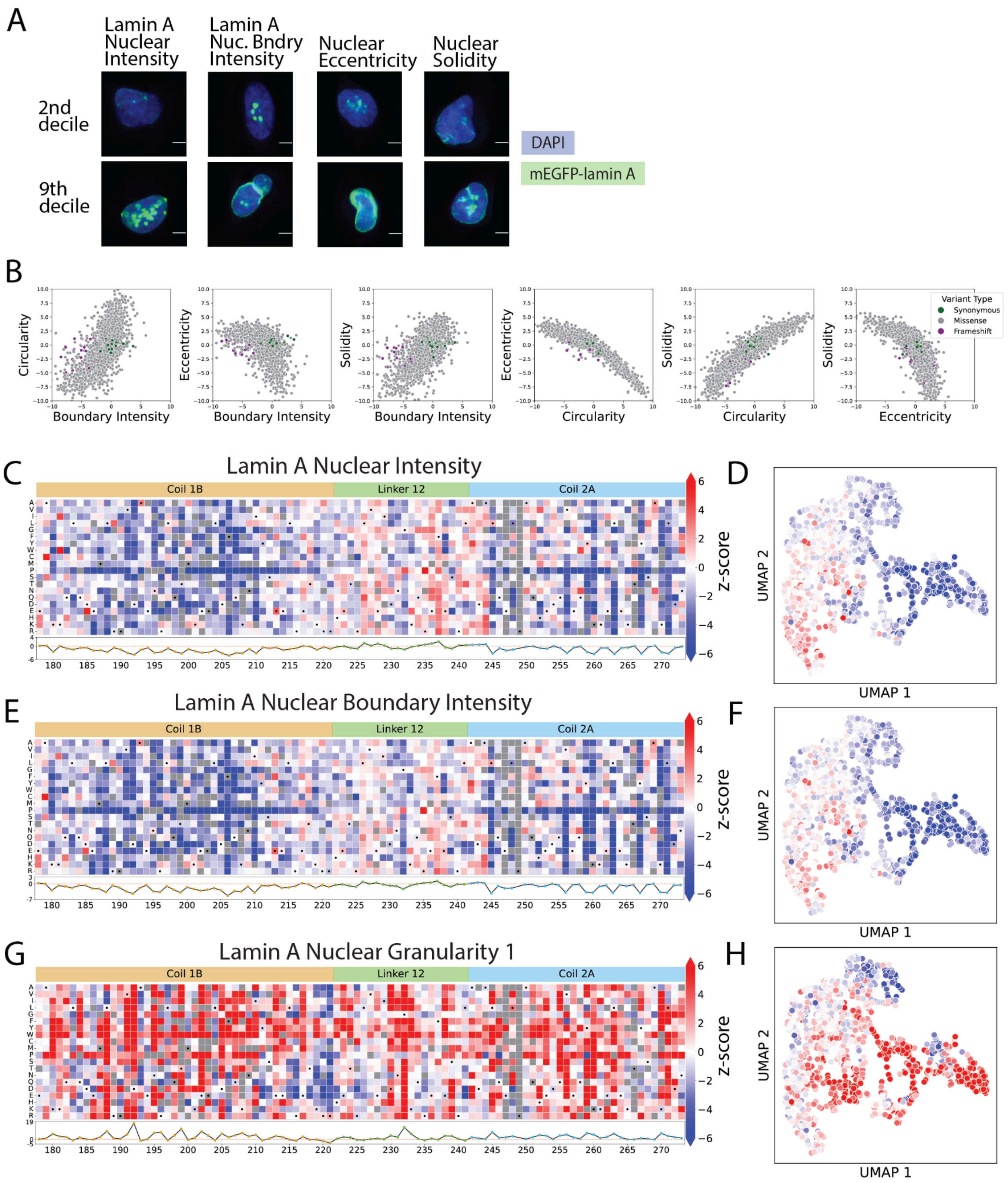

Supplementary Figure 4: *LMNA* VIS-seq landmark feature heatmaps

(A) Randomly-selected cells from the second and ninth deciles in lamin A nuclear intensity, lamin A nuclear boundary intensity, nuclear eccentricity and nuclear solidity scores are shown. mEGFP-tagged lamin A channel is shown in green, and DAPI in blue. Scale bar indicates 5 µm. (B) *LMNA* feature z-scores for mEGFP-lamin A boundary intensity, nuclear eccentricity, nuclear solidity, and nuclear eccentricity are plotted against each other for all profiled variants. Z-scores

bioRxiv preprint doi: https://doi.org/10.1101/2025.07.03.663081; this version posted July 5, 2025. The copyright holder for this preprint (which was not certified by peer review) is the author/funder, who has granted bioRxiv a license to display the preprint in perpetuity. It is made available under aCC-BY 4.0 International license.

are versus the synonymous variant distribution. Variant type is colored according to: synonymous variants (green), missense (grey), and frameshift variants (purple).
(C) Heatmap for missense substitution effects on *LMNA* variant nuclear intensity z-scores. Grey boxes indicate missing variants and boxes with black dots indicate synonymous substitutions. Blue indicates low and red indicates high z-score for feature. *LMNA* subdomains are shown above the heatmap, and position-averaged z-score are plotted below the heatmap.
(D) Lamin A nuclear intensity z-scores plotted on UMAP visualization of *LMNA* variant profiles, colored as in (C).
(E) Heatmap for missense substitution effects on (median over cells) *LMNA* nuclear boundary intensity scores, colored and annotated as in (C).
(F) Lamin A nuclear boundary intensity z-scores plotted on UMAP visualization of *LMNA* variant profiles, colored as in (C).
(G) Heatmap for missense substitution effects on lamin A nuclear granularity 1 z-scores, colored and annotated as in (C).
(H) Lamin A nuclear granularity 1 z-scores plotted on UMAP visualization of *LMNA* variant profiles, colored as in (C).

bioRxiv preprint doi: https://doi.org/10.1101/2025.07.03.663081; this version posted July 5, 2025. The copyright holder for this preprint (which was not certified by peer review) is the author/funder, who has granted bioRxiv a license to display the preprint in perpetuity. It is made available under aCC-BY 4.0 International license.

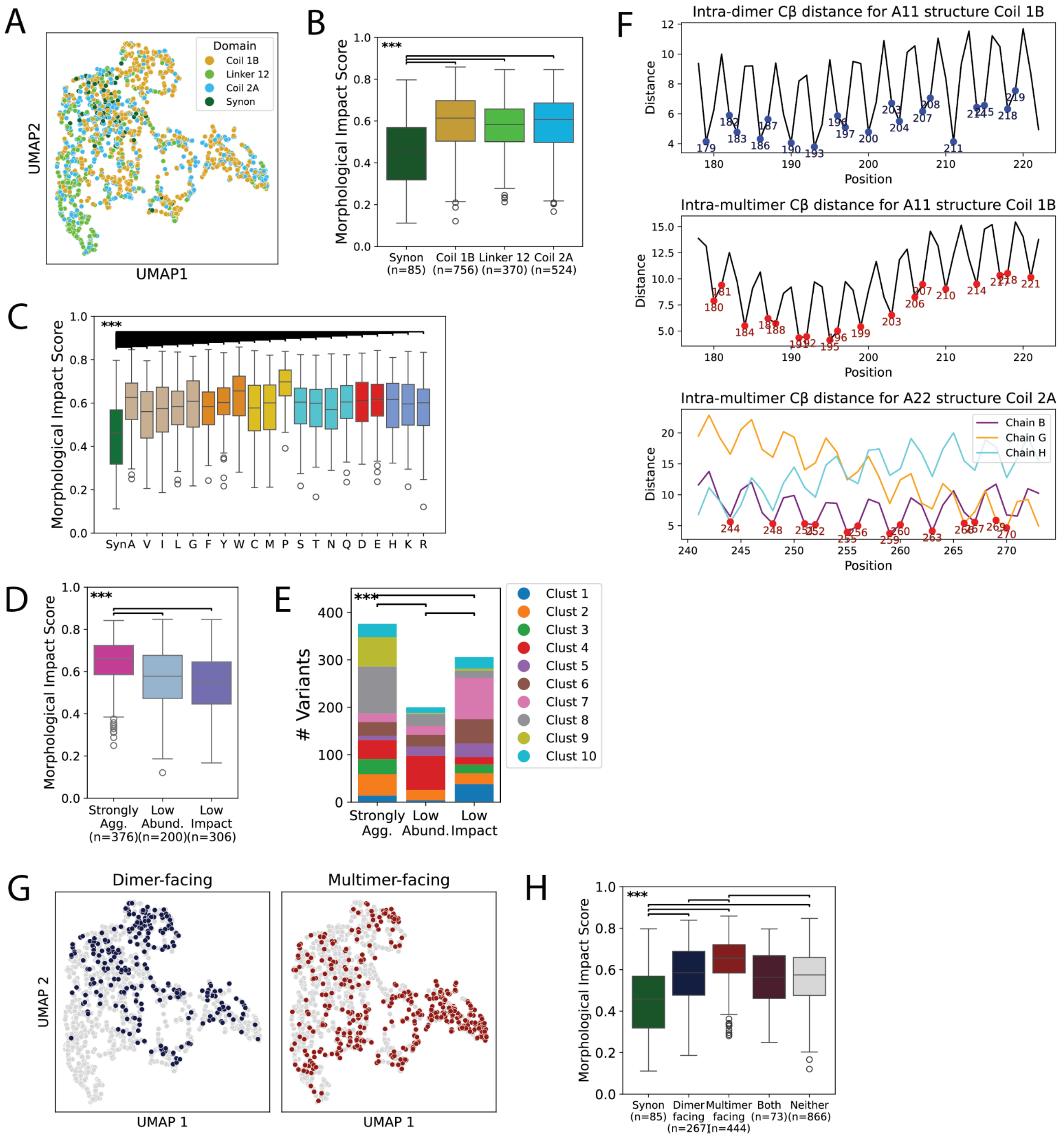

Supplementary Figure 5: VIS-seq profiles separate lamin A residues by structural and functional properties

(A) UMAP visualization of *LMNA* synonymous (green) or missense variant profiles colored by lamin A domain.

(B) Morphological impact score of missense substitutions plotted by domain, compared with synonymous variants (green). *** indicates Mann-Whitney U-test p<0.001.

(C) Morphological impact score of lamin A missense substitutions plotted by amino acid, compared with synonymous variants (green). *** indicates Mann-Whitney U-test p<0.001

bioRxiv preprint doi: https://doi.org/10.1101/2025.07.03.663081; this version posted July 5, 2025. The copyright holder for this preprint (which was not certified by peer review) is the author/funder, who has granted bioRxiv a license to display the preprint in perpetuity. It is made available under aCC-BY 4.0 International license.

(D) Morphological impact score of missense substitutions at α-helical position groups as defined in Fig. 3F by clustering amino acid positions. *** indicates Mann-Whitney U-test $p<0.001$.

(E) Profiled α-helical position variants by position cluster, colored by their Louvain cluster of residence as shown on UMAP representation in Fig. 2H. *** indicates χ^2 $p<0.001$.

(F) Intra-dimer (top) and inter-dimer coil 1B (middle) and coil 2A (bottom) minimum beta-carbon distances derived from A11[63] and A22[64] multimer structures. Dimer-facing residues are indicated in blue (top) and multimer-facing residues are indicated in red for A11[63] and A22[64] structures (middle, bottom, respectively). See Methods for how these residues are defined.

(G) missense substitutions at dimer-facing and multimer-facing residues (as defined in (F)) plotted on the UMAP.

(H) Morphological impact score of synonymous variants (green) and missense substitutions at dimer-facing positions (blue), multimer-facing positions (red), both (purple) and other non-interacting positions (gray). *** indicates Mann-Whitney U-test $p<0.001$.

bioRxiv preprint doi: https://doi.org/10.1101/2025.07.03.663081; this version posted July 5, 2025. The copyright holder for this preprint (which was not certified by peer review) is the author/funder, who has granted bioRxiv a license to display the preprint in perpetuity. It is made available under aCC-BY 4.0 International license.

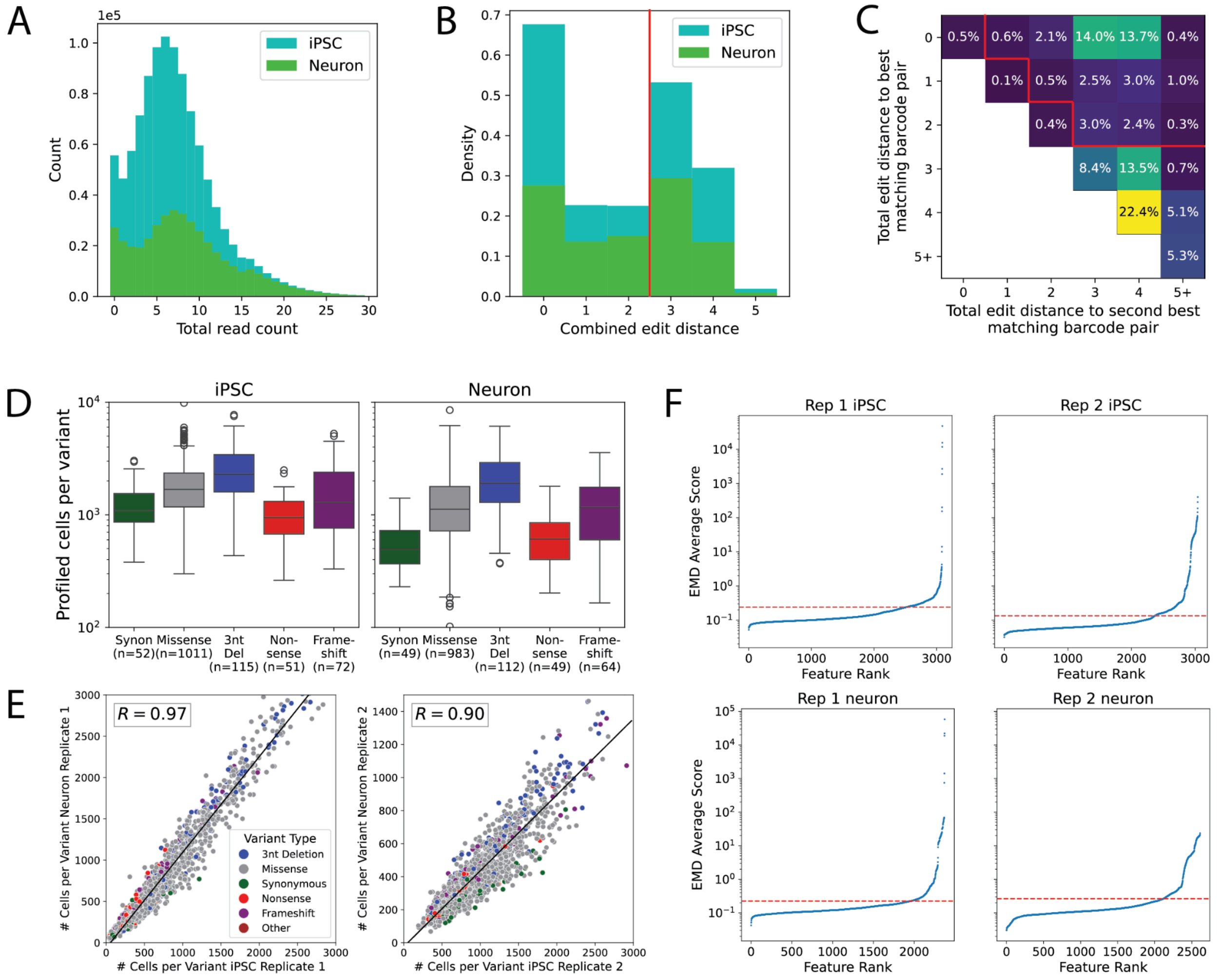

Supplementary Figure 6: VIS-seq reproducibly called *PTEN* variants in single cells in iPS cells and neurons from two 8bp barcodes

(A) Histogram of total number of 8-base pair reads per genotyped cell in single well of *PTEN* iPS cell (blue) or neuron (green) replicate 2 experiments.

(B) Total (summed) edit distance between consensus cell-level 16-base pair double barcode reads and nearest library double barcode in a single well of *PTEN* iPS cell (blue) or neuron (green) replicate 2 experiments. Red line indicates that cells with total edit distance < 3 were used if they matched to a unique library double barcode.

(C) Total distance between consensus cell-level 16-base pair double barcode reads and nearest library double barcode (x) or second nearest library double barcode (y) in a single well of *PTEN* iPS cell replicate 2 experiment. Red line indicates that cells with total edit distance < 3 were used if they matched to a unique library double barcode.

(D) Numbers of profiled single iPS cells (left) or neurons (right) containing single *PTEN* variants over the two replicate experiments are plotted by variant type.

(E) Number of cells genotyped for each *PTEN* variant in iPSC (x) and neuron (y) of replicate 1 (left) and replicate 2 (right) of VIS-seq screens, with Pearson's r shown.

(F) EMD reproducibility scores derived from 30 random partitions of wild-type *PTEN* expressing iPS cells (top) or neurons (bottom) ranked by feature for replicate 1 (left) and replicate 2 (right).

bioRxiv preprint doi: https://doi.org/10.1101/2025.07.03.663081; this version posted July 5, 2025. The copyright holder for this preprint (which was not certified by peer review) is the author/funder, who has granted bioRxiv a license to display the preprint in perpetuity. It is made available under aCC-BY 4.0 International license.

Low scores indicate high reproducibility. Threshold is drawn at 1.5 times the IQR added to the first quartile. Feature EMDs above this threshold in either replicate were removed in the feature selection step.

bioRxiv preprint doi: https://doi.org/10.1101/2025.07.03.663081; this version posted July 5, 2025. The copyright holder for this preprint (which was not certified by peer review) is the author/funder, who has granted bioRxiv a license to display the preprint in perpetuity. It is made available under aCC-BY 4.0 International license.

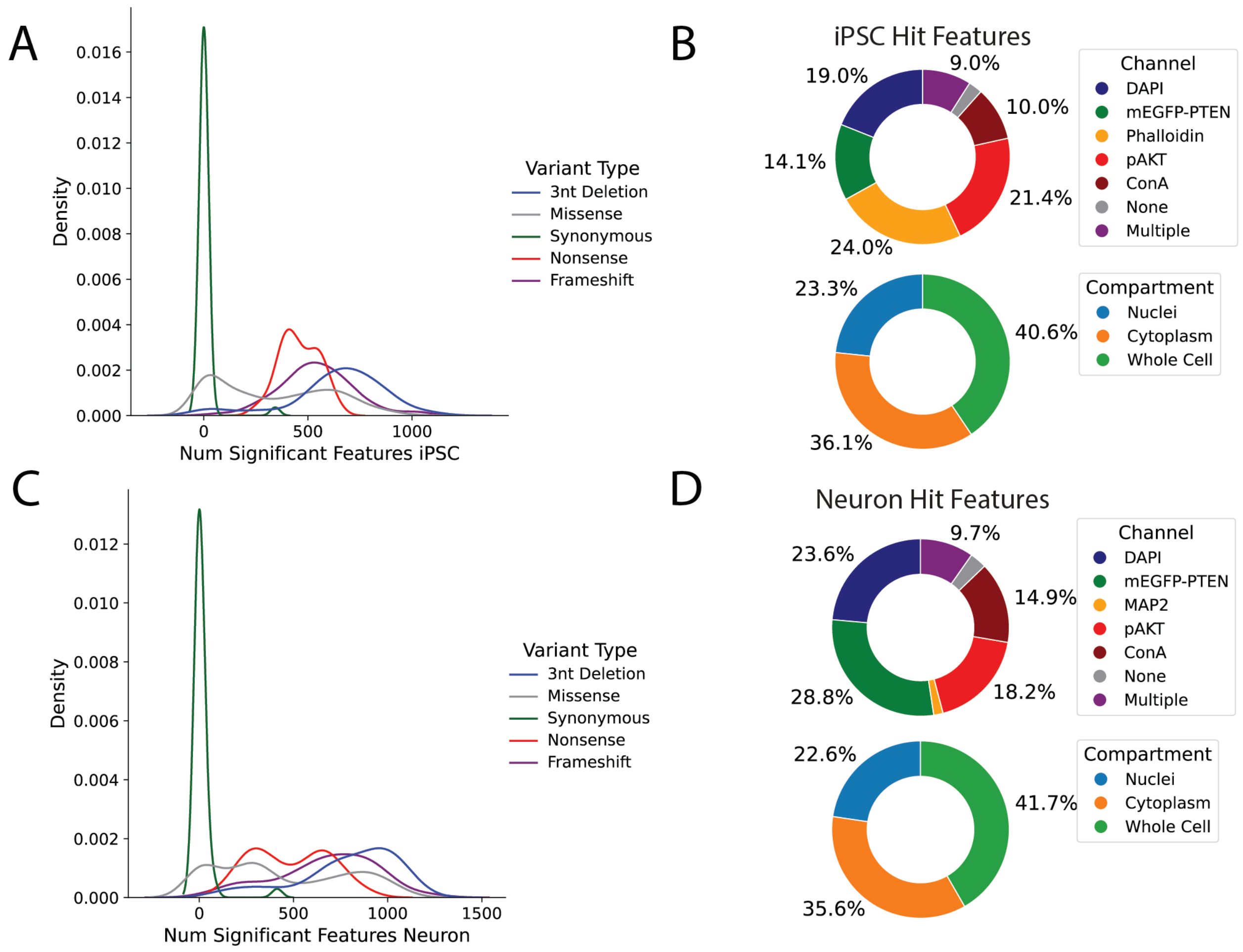

Supplementary Figure 7: Significant features in VIS-seq *PTEN* experiments

(A) Number of significant features in iPSC for each variant class determined by KS-test against wild-type cells. Significance indicates Bonferroni-corrected p<0.001.

(B) Hit iPS cell features, defined as being significantly different from WT (at Bonferroni-corrected KS-test p<0.001) in >=25 *PTEN* variants in iPS cells, are classified by imaging channel (top) or by compartment (bottom).

(C) Number of significant features in neurons for each variant class determined by KS-test against wild-type cells. Significance indicates Bonferroni-corrected p<0.001.

(D) Hit neuron features, defined as being significantly different from WT (at Bonferroni-corrected KS-test p<0.001) in >=25 *PTEN* variants in neurons, are classified by imaging channel (top) or by compartment (bottom).

bioRxiv preprint doi: https://doi.org/10.1101/2025.07.03.663081; this version posted July 5, 2025. The copyright holder for this preprint (which was not certified by peer review) is the author/funder, who has granted bioRxiv a license to display the preprint in perpetuity. It is made available under aCC-BY 4.0 International license.

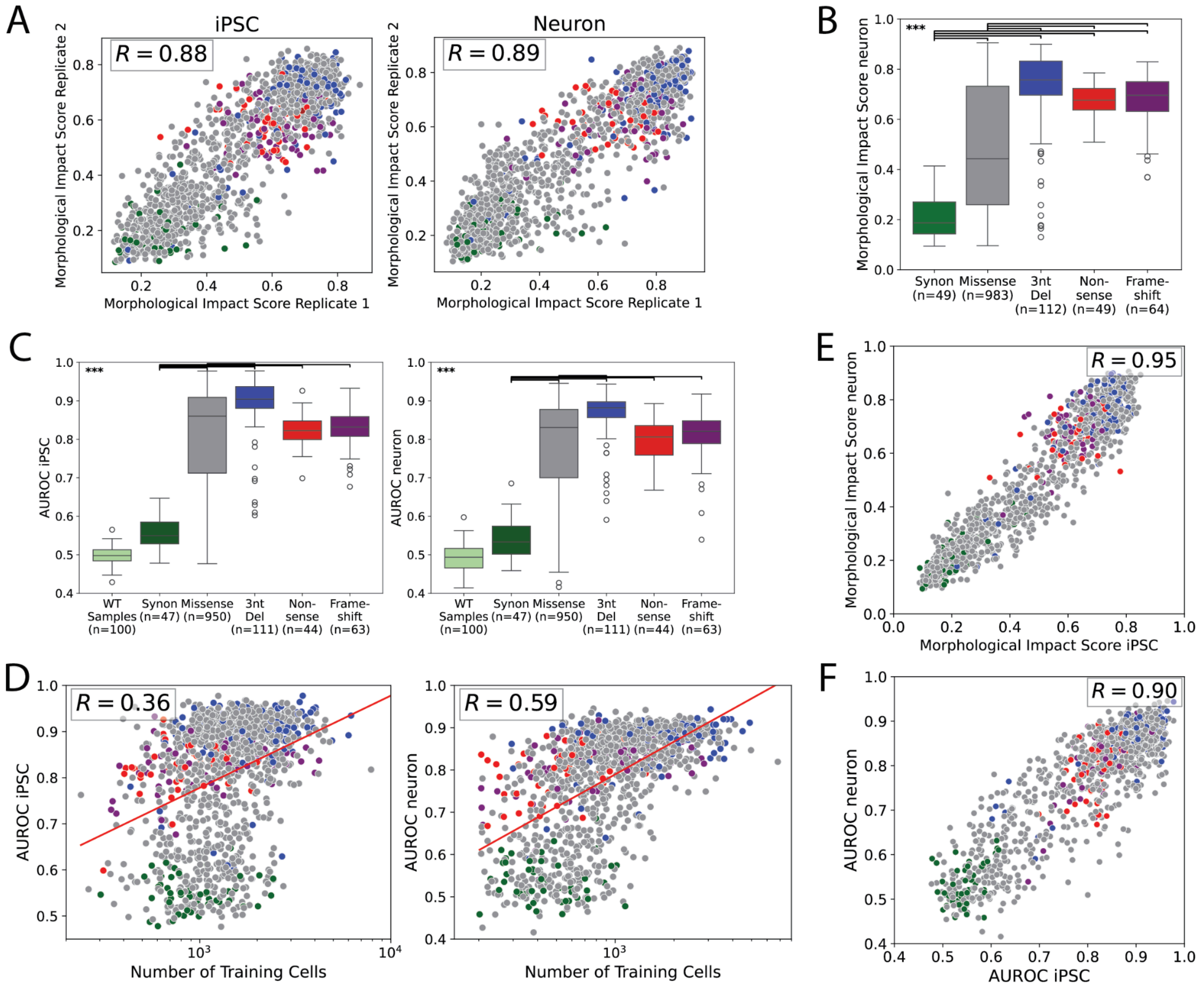

Supplementary Figure 8: VIS-seq generated reproducible profiles in iPS cells and neurons

(A) Morphological impact scores for variants in each replicate of *PTEN* VIS-seq screen in iPSC (left) and neurons (right), colored by variant type as in (B), with Pearson's r shown.

(B) Morphological impact score for *PTEN* variant profiles in neurons, plotted by variant type. *** indicates Mann-Whitney p-value < 0.001.

(C) AUROC scores for *PTEN* variants in iPS cells (left) or neurons (right), plotted by variant type. 100 bootstrapped samples of WT variants are also shown for comparison. *** indicates Mann-Whitney p-value < 0.001.

(D) Scatterplot showing the AUROC score for each variant against the number of training iPS cells (left) or neurons (right) used for that variant. Variants are colored by variant type as in (B). Best fit line and Pearson's r shown.

(E) Morphological impact scores for *PTEN* variants in both iPSC (x) and neurons (y) are compared, colored by variant type as in (B). Pearson's r shown.

(F) AUROC scores for *PTEN* variants in both iPSC (x) and neurons (y) are compared, colored by variant type as in (B). Pearson's r shown.

bioRxiv preprint doi: https://doi.org/10.1101/2025.07.03.663081; this version posted July 5, 2025. The copyright holder for this preprint (which was not certified by peer review) is the author/funder, who has granted bioRxiv a license to display the preprint in perpetuity. It is made available under aCC-BY 4.0 International license.

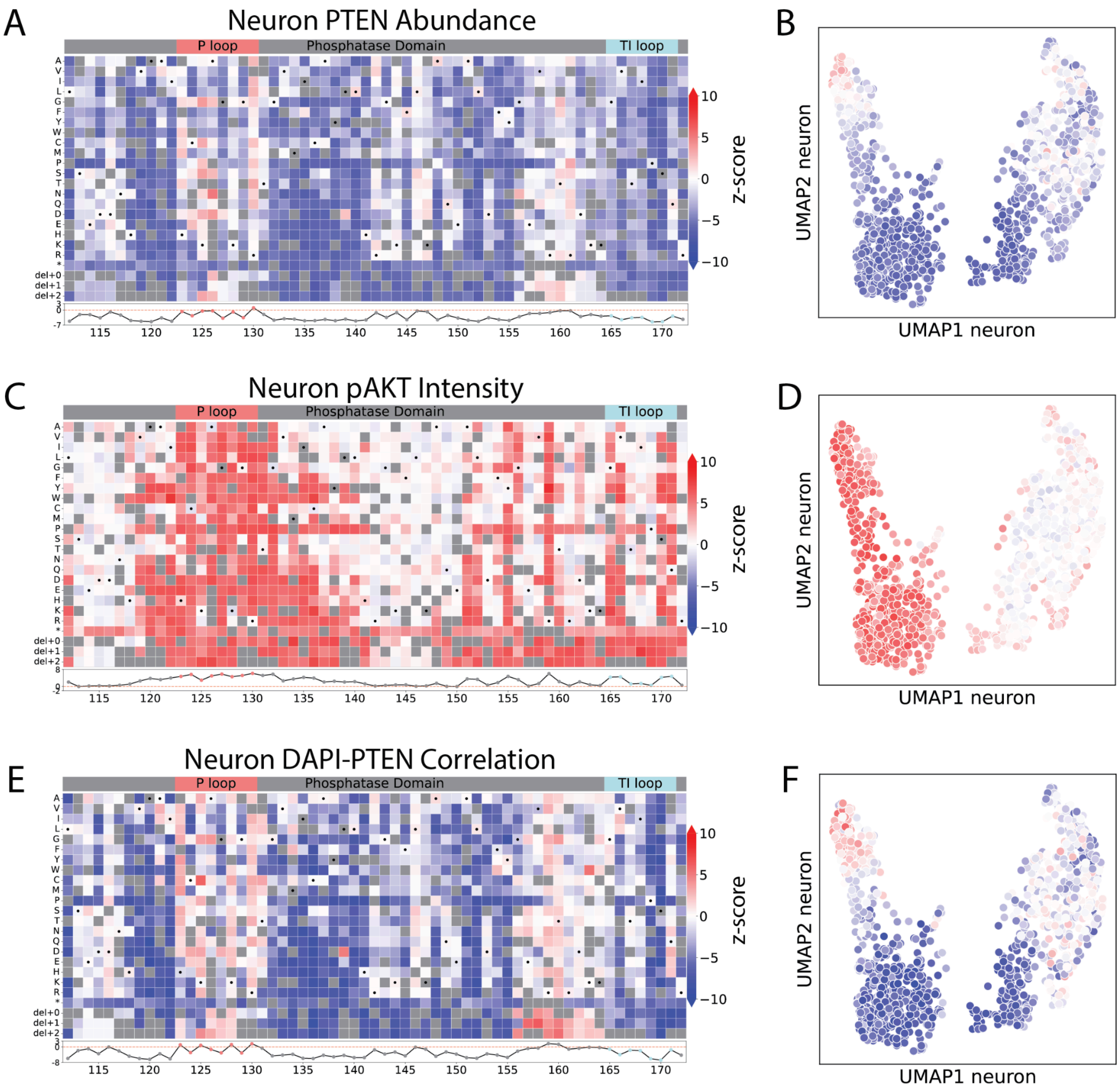

Supplementary Figure 9: *PTEN* landmark features in neurons

(A) Positional heatmap (top) of PTEN intensity scores for missense, stop gain, or 3-nucleotide deletion variant profiles in neurons. Positional average of scores (bottom) with P-loop and TI-loop residues highlighted.

(B) UMAP of *PTEN* variant morphological profiles in neurons colored by PTEN intensity score. Blue indicates low and red indicates high synonymous z-score for feature, colored as in (A).

(C) Positional heatmap (top) of pAKT intensity scores for missense, stop gain, or 3-nucleotide deletion variant profiles in neurons. Positional average of scores (bottom) with P-loop and TI-loop residues highlighted.

(D) UMAP of *PTEN* variant morphological profiles in neurons colored by pAKT intensity z-score (right). Blue indicates low and red indicates high synonymous z-score for feature, colored as in (C).

bioRxiv preprint doi: https://doi.org/10.1101/2025.07.03.663081; this version posted July 5, 2025. The copyright holder for this preprint (which was not certified by peer review) is the author/funder, who has granted bioRxiv a license to display the preprint in perpetuity. It is made available under aCC-BY 4.0 International license.

(E) Positional heatmap (top) of DAPI-PTEN correlation scores for missense, stop gain, or 3-nucleotide deletion variant profiles in neurons. Positional average of scores (bottom) with P-loop and TI-loop residues highlighted.
(F) UMAP of *PTEN* variant morphological profiles in neurons colored by DAPI-PTEN correlation. Blue indicates low and red indicates high synonymous *z*-score for feature, colored as in (E).

bioRxiv preprint doi: https://doi.org/10.1101/2025.07.03.663081; this version posted July 5, 2025. The copyright holder for this preprint (which was not certified by peer review) is the author/funder, who has granted bioRxiv a license to display the preprint in perpetuity. It is made available under aCC-BY 4.0 International license.

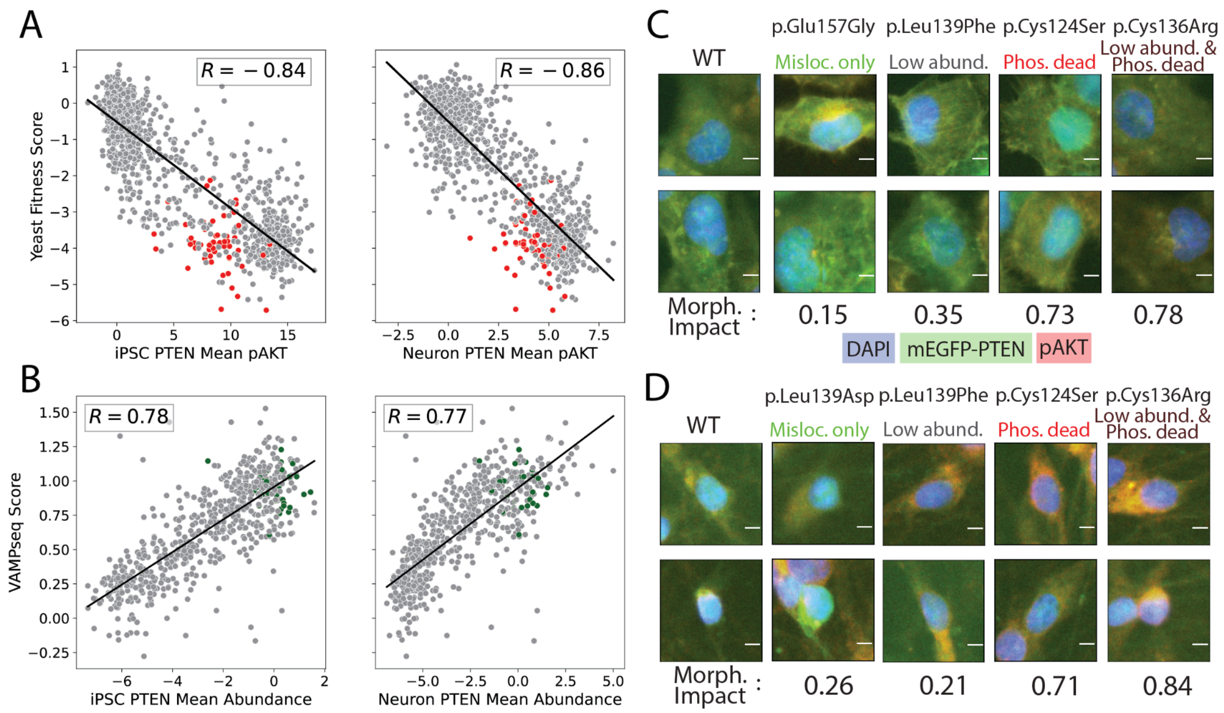

Supplementary Figure 10: *PTEN* VIS-seq measurements correlate with prior variant effect measurements

(A) pAKT intensity score for *PTEN* variants in iPSC (left) and neurons (right) plotted against yeast fitness score, with best-fit line and Pearson's r shown.

(B) mEGFP-PTEN abundance score for *PTEN* variants in iPSC (left) and neurons (right) plotted against VAMP-seq score, with best-fit line and Pearson's r shown.

(C) Two randomly-selected iPS cells expressing *PTEN* variants are shown, with corresponding morphological impact scores. mEGFP-tagged PTEN channel is shown in green, DAPI in blue, and pAKT in red. Scale bar indicates 5 μm.

(D) Two randomly-selected neurons expressing *PTEN* variants are shown, with corresponding morphological impact scores. mEGFP-tagged PTEN channel is shown in green, DAPI in blue, and pAKT in red. Scale bar indicates 5 μm.

bioRxiv preprint doi: https://doi.org/10.1101/2025.07.03.663081; this version posted July 5, 2025. The copyright holder for this preprint (which was not certified by peer review) is the author/funder, who has granted bioRxiv a license to display the preprint in perpetuity. It is made available under aCC-BY 4.0 International license.

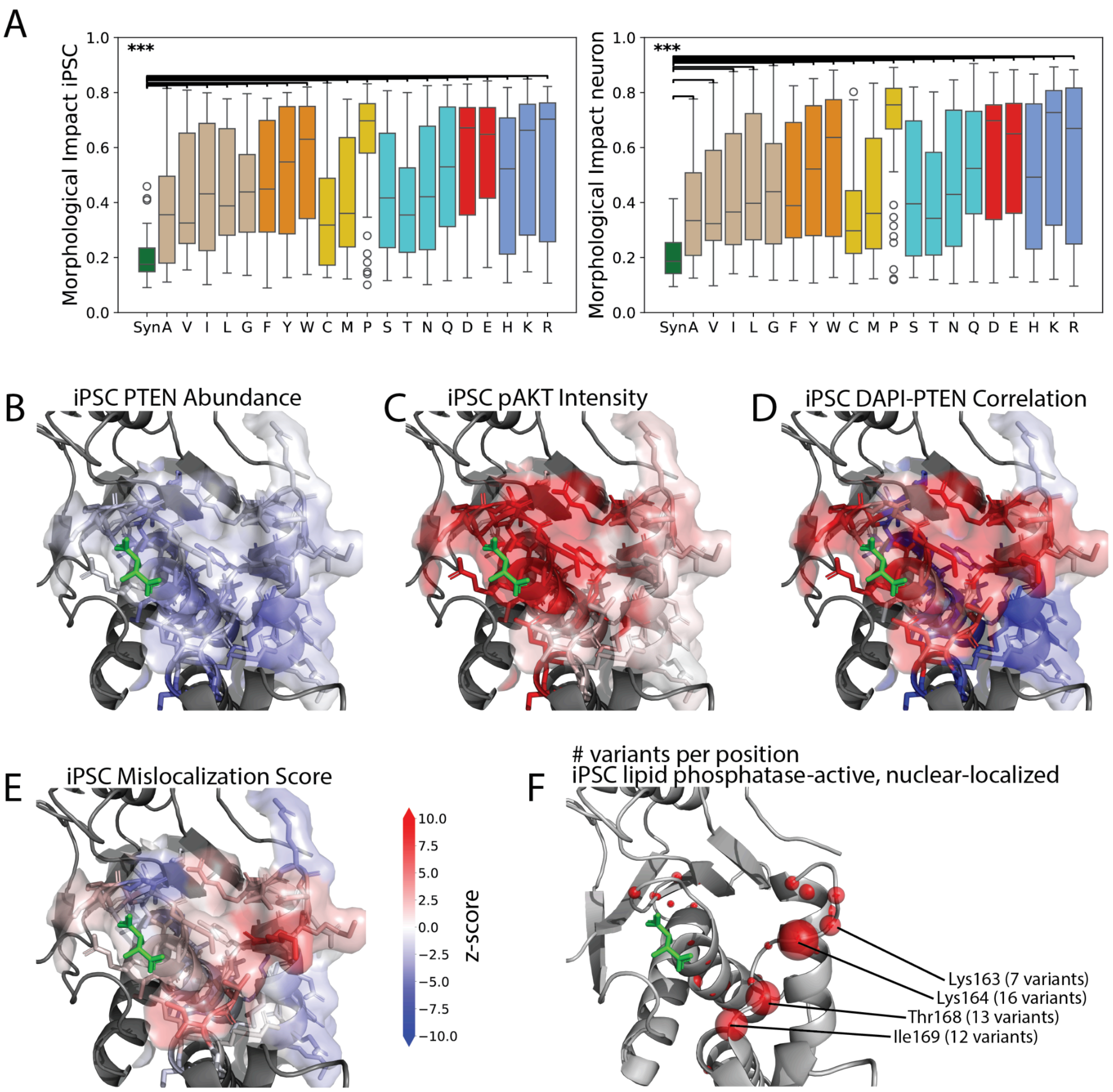

Supplementary Figure 11: Structural analysis of *PTEN* VIS-seq profiles

(A) Morphological impact score of missense substitutions plotted by amino acid, compared with synonymous variants, in iPSC (left) and neurons (right). *** indicates Mann-Whitney U-test p<0.001

(B) iPSC mEGFP-PTEN intensity score positional averages are used to color the PTEN crystal structure (1D5R)[69]. Solvent-exposed surface is colored by the exposed residue's z-score. Blue indicates low-intensity positions. The bound tartrate molecule is shown in green. Residue color scale shown to the right of (E).

(C) iPSC pAKT intensity score positional averages are used to color the PTEN crystal structure (1D5R)[69]. Red indicates positions with high pAKT intensity and corresponding low lipid phosphatase activity. Residue color scale shown to the right of (E).

bioRxiv preprint doi: https://doi.org/10.1101/2025.07.03.663081; this version posted July 5, 2025. The copyright holder for this preprint (which was not certified by peer review) is the author/funder, who has granted bioRxiv a license to display the preprint in perpetuity. It is made available under aCC-BY 4.0 International license.

(D) iPSC DAPI-PTEN correlation score positional averages are used to color the PTEN crystal structure (1D5R)[69]. Blue indicates cytoplasmic-localizing positions and red indicates nuclear-localizing positions. Residue color scale shown to the right of (E).
(E) iPSC mislocalization score (see Methods) positional averages are used to color the PTEN crystal structure (1D5R)[69]. Blue indicates aberrantly cytoplasmic-localizing positions and red indicates aberrantly nuclear-localizing positions, after the effects of activity and abundance are removed. Residue color scale shown to the right.
(F) PTEN structure (1D5R)[69] with spheres centered at α-carbon atoms with radii indicating the number of lipid-phosphatase active (defined as pAKT z-score<2.5) and nuclear-localized (defined as DAPI-PTEN correlation z-score>2.5) variants at that position. The top four positions indicating nuclear localization are highlighted, with the number of variants at each of these positions indicated.

bioRxiv preprint doi: https://doi.org/10.1101/2025.07.03.663081; this version posted July 5, 2025. The copyright holder for this preprint (which was not certified by peer review) is the author/funder, who has granted bioRxiv a license to display the preprint in perpetuity. It is made available under aCC-BY 4.0 International license.

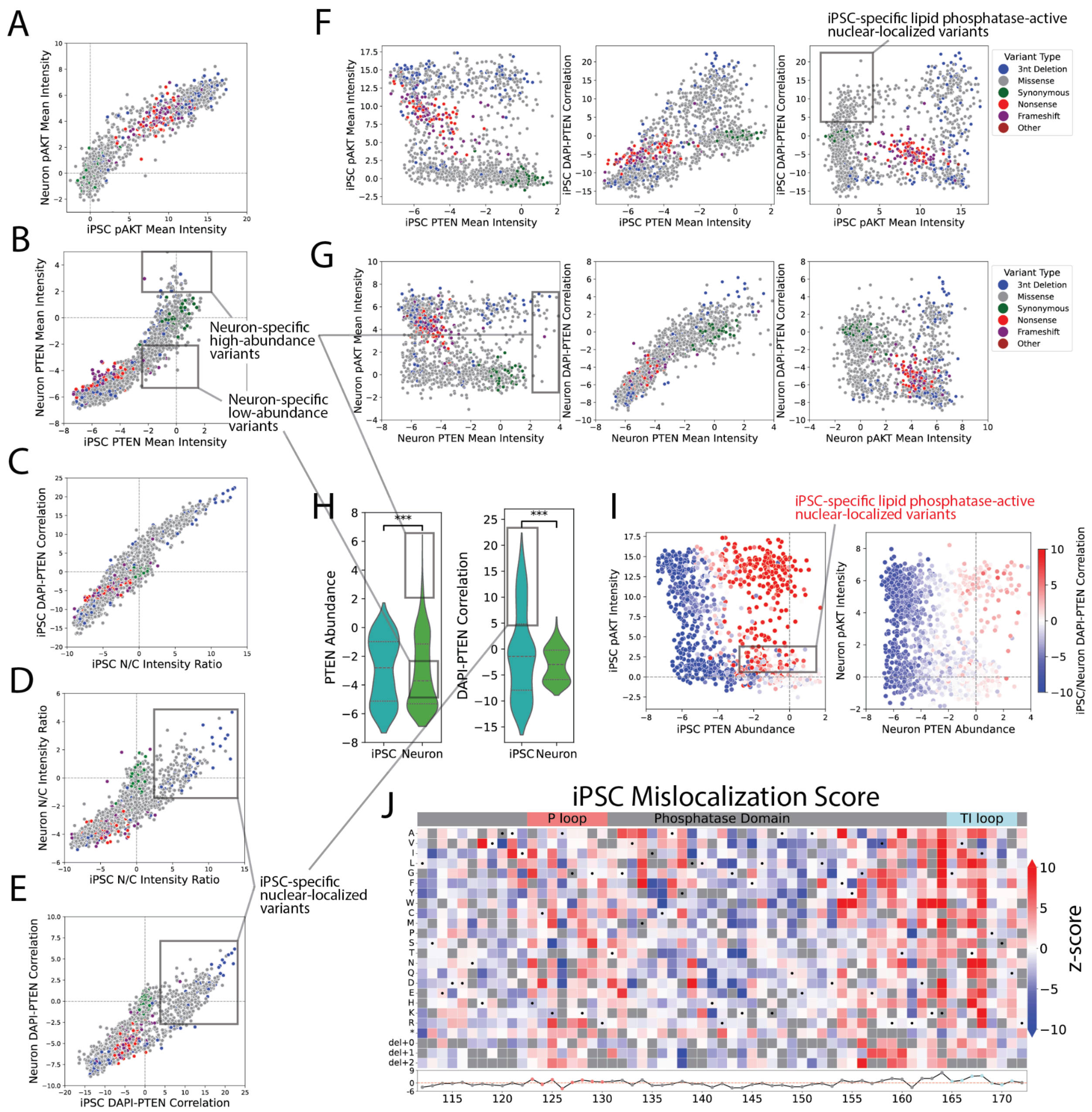

Supplementary Figure 12: Relationships between *PTEN* landmark features in iPS cells and neurons

(A) iPSC pAKT intensity z-scores are plotted against neuron pAKT intensity z-scores for all profiled variant, colored by variant type as in (F). Z-scores are versus the synonymous variant distribution.

(B) iPSC mEGFP-PTEN intensity z-scores are plotted against neuron mEGFP-PTEN intensity z-scores for all profiled variants, colored by variant type as in (F). Both neuron-specific low- and high-abundance variants are indicated.

(C) iPSC nucleus to cytoplasm PTEN intensity ratio z-scores are plotted against iPSC DAPI-PTEN correlation z-scores for all profiled variants, colored by variant type as in (F).

bioRxiv preprint doi: https://doi.org/10.1101/2025.07.03.663081; this version posted July 5, 2025. The copyright holder for this preprint (which was not certified by peer review) is the author/funder, who has granted bioRxiv a license to display the preprint in perpetuity. It is made available under aCC-BY 4.0 International license.

(D) iPSC nucleus to cytoplasm PTEN intensity ratio z-scores are plotted against neuron nucleus to cytoplasm PTEN intensity ratio z-scores, colored by variant type as in (F). iPSC-specific nuclear-localized variants are indicated.
(E) iPSC DAPI-PTEN correlation z-scores are plotted against neuron DAPI-PTEN correlation z-scores for all profiled variants, colored by variant type as in (F). iPSC-specific nuclear-localized variants are indicated.
(F) iPSC PTEN landmark feature z-scores (PTEN intensity, pAKT intensity, and DAPI-PTEN correlation) are plotted against each other for all profiled variants. Variant type is colored according to: synonymous variants (green), missense (grey), 3-nt deletions (blue), nonsense (red), and frameshift variants (purple). iPSC-specific lipid phosphatase-active nuclear-localized variants are indicated.
(G) Neuron PTEN landmark feature z-scores are plotted against each other for all profiled variant, colored by variant type as in (F). Neuron-specific high-abundance variants are indicated.
(H) Violin plots of iPS cell and neuron PTEN abundance (left) and DAPI-PTEN correlation z-scores (right). Neuron-specific low- and high-abundance variants and iPSC-specific nuclear-localized variants are indicated. *** indicates KS p-value<0.001.
(I) iPSC (left) or neuron (right) PTEN abundance z-scores are plotted against pAKT intensity z-scores, colored by either iPSC DAPI-PTEN correlation (left) or neuron DAPI-PTEN correlation (right) z-scores. iPSC-specific phosphatase-active, nuclearly-localized variants are indicated on the iPSC plot, as red-colored variants (DAPI-PTEN correlation z-score > 2.5) with pAKT intensity z-score < 2.5.
(J) The mislocalization score is defined as the variant-level residuals when DAPI-PTEN correlation is regressed against PTEN intensity and pAKT intensity (see Methods). Positional heatmap (top) of mislocalization scores for missense, stop gain, or 3-nucleotide deletion variant profiles in iPSC. Positional average of scores (bottom) with P-loop and TI-loop residues highlighted. Blue indicates low score and red indicates high score.

bioRxiv preprint doi: https://doi.org/10.1101/2025.07.03.663081; this version posted July 5, 2025. The copyright holder for this preprint (which was not certified by peer review) is the author/funder, who has granted bioRxiv a license to display the preprint in perpetuity. It is made available under aCC-BY 4.0 International license.

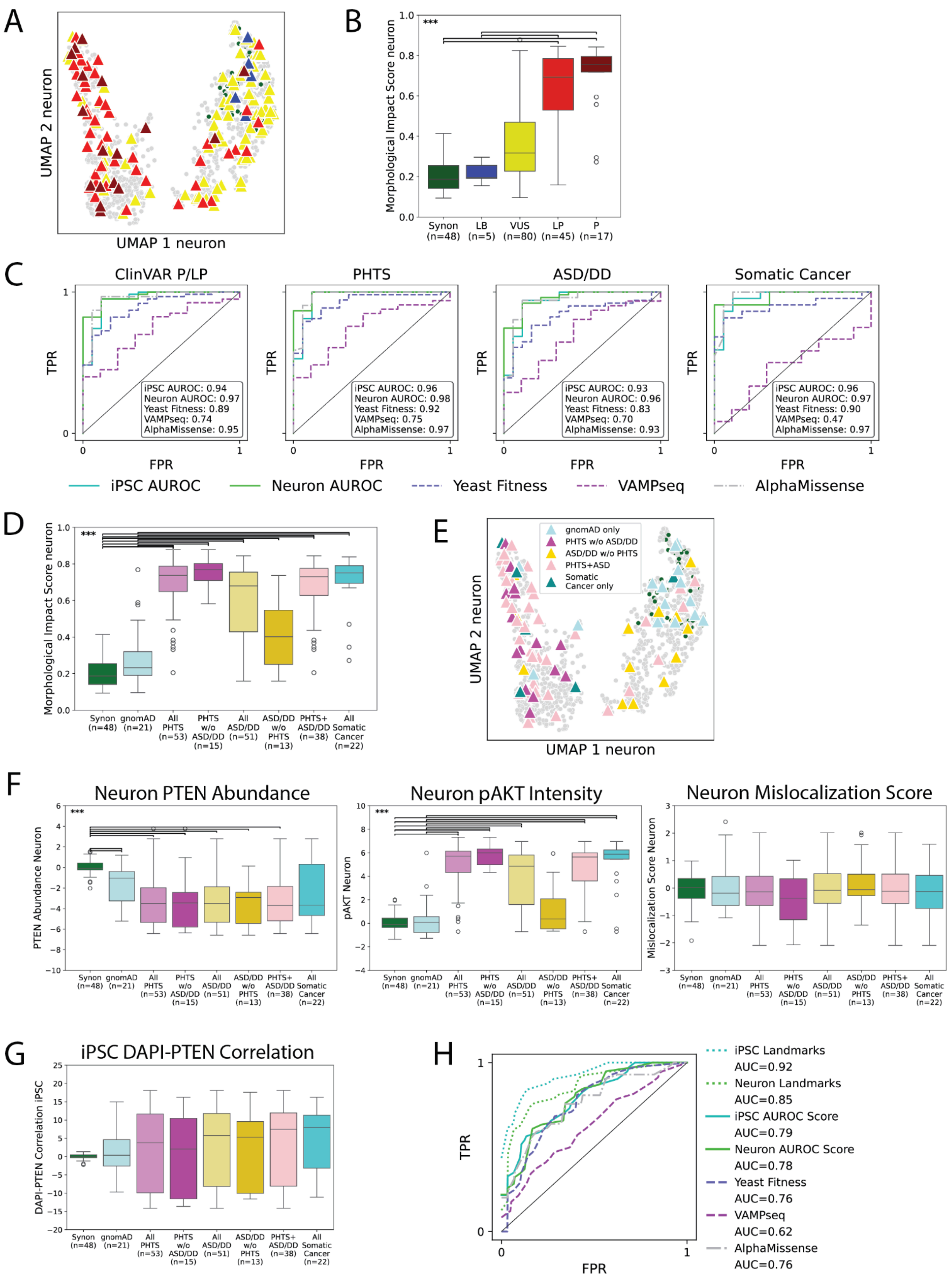

bioRxiv preprint doi: https://doi.org/10.1101/2025.07.03.663081; this version posted July 5, 2025. The copyright holder for this preprint (which was not certified by peer review) is the author/funder, who has granted bioRxiv a license to display the preprint in perpetuity. It is made available under aCC-BY 4.0 International license.

Supplementary Figure 13: *PTEN* VIS-seq profiles predict pathogenicity and clinical phenotype

(A) UMAP visualization of neuron *PTEN* variant profiles. Triangles indicate variants in ClinVar classified as likely benign (LB, blue), likely pathogenic (LP, red), pathogenic (P, dark red), or variant of uncertain significance (VUS, yellow). All profiled variants are plotted in the background colored green (synonymous) or grey (otherwise) for comparison.

(B) Morphological impact scores for *PTEN* neuron profiles are plotted by ClinVar label. Synonymous variants (green) are included for comparison. *** indicates Mann-Whiney U p<0.001.

(C) Receiver operating characteristic (ROC) curves are plotted for univariate zero-shot models predicting ClinVar pathogenicity or each clinical phenotype (ASD = autism spectrum disorder, DD = developmental delay, PHTS = *PTEN* hamartoma tumor syndrome; see Methods for curation criteria) from iPSC and neuron AUROC score (this publication, solid lines), yeast fitness scores[83] (dashed line), VAMPseq scores[5] (dashed line), or AlphaMissense scores[91] (dot-dashed line). Area under the curve (AUC) scores are shown in the box for each model.

(D) Morphological impact scores for *PTEN* neuron profiles are plotted by variant association with clinical phenotypes. gnomAD v4.1 (light blue) variants and synonymous variants (green) are plotted for comparison. *** indicates Mann-Whiney U p<0.001.

(E) UMAP visualization of iPS cell *PTEN* variant profiles. Triangles indicate association with clinical phenotypes. gnomAD v4.1 (light blue) variants are also plotted. All profiled variants are plotted in the background colored green (synonymous) or grey (otherwise) for comparison.

(F) Feature scores for *PTEN* variants in neurons are plotted by variant association with clinical phenotypes. gnomAD v4.1 (light blue) variants and synonymous variants (green) are plotted for comparison. Features plotted include PTEN intensity (left), pAKT intensity (center) and mislocalization score (right, see Methods).*** indicates Mann-Whitney U-test p<0.001

(G) Feature scores for *PTEN* variants in iPSC are plotted by variant association with landmark feature iPS cell DAPI-PTEN correlation z-scores relative to the synonymous variant distribution. gnomAD v4.1 (light blue) variants and synonymous variants (green) are plotted for comparison.

(H) Receiver operating characteristic (ROC) curves produced by macro-averaging sensitivity and specificity over classes for models trained on iPS and neuron VIS-seq landmark features (dotted lines) as well as scores from (C) classifying gnomAD controls from PHTS-associated variants from ASD/DD-associated variants. AUC is shown on the right for each model.

bioRxiv preprint doi: https://doi.org/10.1101/2025.07.03.663081; this version posted July 5, 2025. The copyright holder for this preprint (which was not certified by peer review) is the author/funder, who has granted bioRxiv a license to display the preprint in perpetuity. It is made available under aCC-BY 4.0 International license.

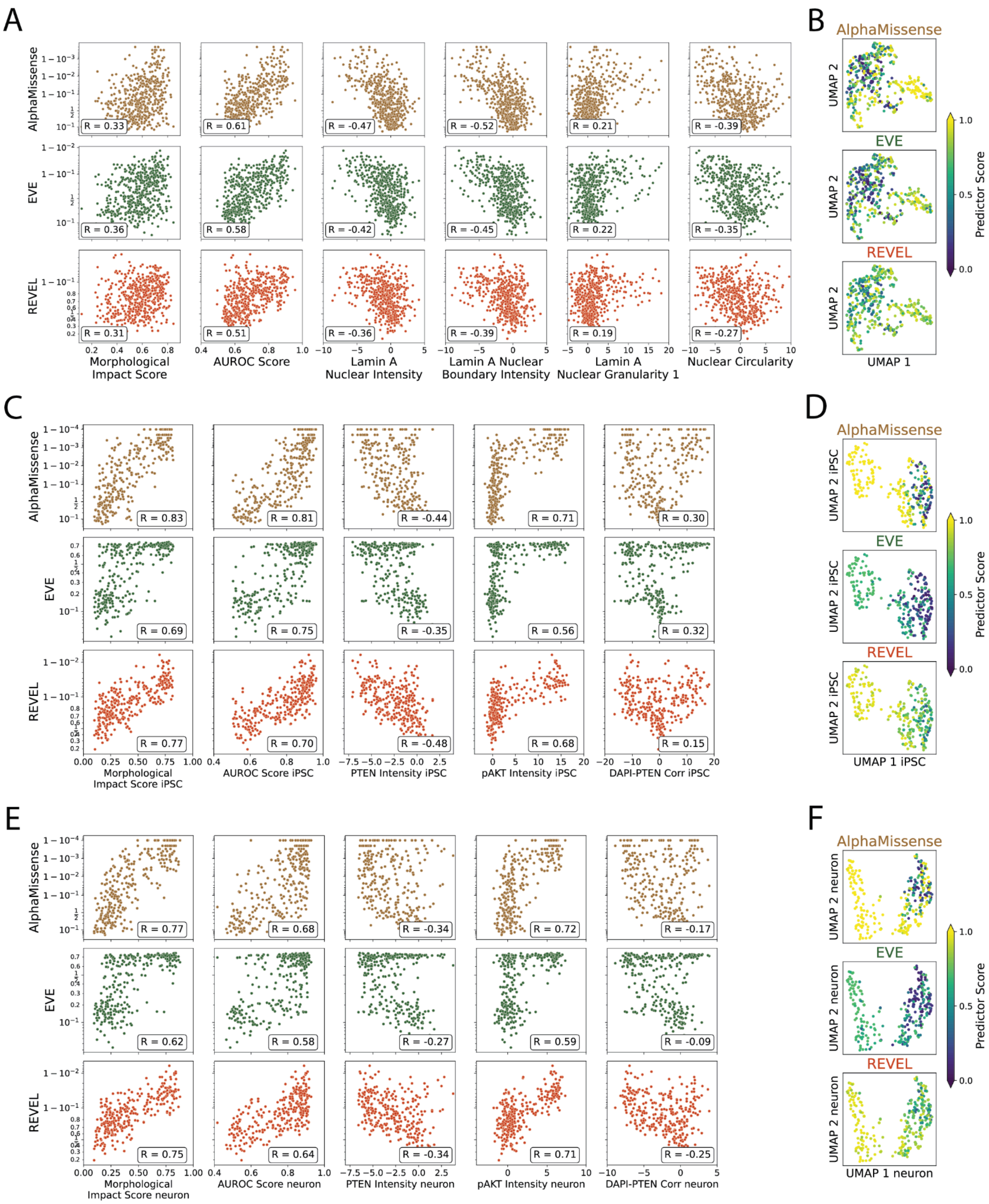

Supplementary Figure 14: VIS-seq profiles elaborate computational variant effect predictions

(A) Predictor scores for AlphaMissense[91], EVE[92], and REVEL[93] are plotted against *LMNA* VIS-seq morphological impact score and landmark features. Pearson's r between logit-transformed predictor scores and VIS-seq scores are shown on the bottom right.

bioRxiv preprint doi: https://doi.org/10.1101/2025.07.03.663081; this version posted July 5, 2025. The copyright holder for this preprint (which was not certified by peer review) is the author/funder, who has granted bioRxiv a license to display the preprint in perpetuity. It is made available under aCC-BY 4.0 International license.

(B) Predictor scores for AlphaMissense[91], EVE[92], and REVEL[93] are colored on the UMAP visualization of *LMNA* VIS-seq profiles.
(C) Predictor scores for AlphaMissense[91], EVE[92], and REVEL[93] are plotted against *PTEN* VIS-seq iPSC morphological impact score and landmark features. Pearson's r between logit-transformed predictor scores and VIS-seq scores are shown on the bottom left.
(D) Predictor scores for AlphaMissense[91], EVE[92], and REVEL[93] are colored on the UMAP visualization of *PTEN* iPSC VIS-seq profiles.
(E) Predictor scores for AlphaMissense[91], EVE[92], and REVEL[93] are plotted against *PTEN* VIS-seq neuron morphological impact score and landmark features. Pearson's r between logit-transformed predictor scores and VIS-seq scores are shown on the bottom left.
(F) Predictor scores for AlphaMissense[91], EVE[92], and REVEL[93] are colored on the UMAP visualization of *PTEN* neuron VIS-seq profiles.
